# Identification and characterisation of the high-risk surgical population in the United Kingdom

**DOI:** 10.1186/cc4928

**Published:** 2006-06-02

**Authors:** Rupert M Pearse, David A Harrison, Philip James, David Watson, Charles Hinds, Andrew Rhodes, R Michael Grounds, E David Bennett

**Affiliations:** 1 William Harvey Research Institute, Queen Mary's School of Medicine and Dentistry, London, UK; 2 Intensive Care National Audit & Research Centre, London, UK; 3 CHKS Ltd, Alcester, Warwickshire, UK; 4 Intensive Care Unit, St George's Hospital, London, UK

## Abstract

**Introduction:**

Little is known about mortality rates following general surgical procedures in the United Kingdom. Deaths are most common in the 'high-risk' surgical population consisting mainly of older patients, with coexisting medical disease, who undergo major surgery. Only limited data are presently available to describe this population. The aim of the present study was to estimate the size of the high-risk general surgical population and to describe the outcome and intensive care unit (ICU) resource use.

**Methods:**

Data on inpatient general surgical procedures and ICU admissions in 94 National Health Service hospitals between January 1999 and October 2004 were extracted from the Intensive Care National Audit & Research Centre database and the CHKS database. High-risk surgical procedures were defined prospectively as those for which the mortality rate was 5% or greater.

**Results:**

There were 4,117,727 surgical procedures; 2,893,432 were elective (12,704 deaths; 0.44%) and 1,224,295 were emergencies (65,674 deaths; 5.4%). A high-risk population of 513,924 patients was identified (63,340 deaths; 12.3%), which accounted for 83.8% of deaths but for only 12.5% of procedures. This population had a prolonged hospital stay (median, 16 days; interquartile range, 9–29 days). There were 59,424 ICU admissions (11,398 deaths; 19%). Among admissions directly to the ICU following surgery, there were 31,633 elective admissions with 3,199 deaths (10.1%) and 24,764 emergency admissions with 7,084 deaths (28.6%). The ICU stays were short (median, 1.6 days; interquartile range, 0.8–3.7 days) but hospital admissions for those admitted to the ICU were prolonged (median, 16 days; interquartile range, 10–30 days). Among the ICU population, 40.8% of deaths occurred after the initial discharge from the ICU. The highest mortality rate (39%) occurred in the population admitted to the ICU following initial postoperative care on a standard ward.

**Conclusion:**

A large high-risk surgical population accounts for 12.5% of surgical procedures but for more than 80% of deaths. Despite high mortality rates, fewer than 15% of these patients are admitted to the ICU.

## Introduction

Reducing mortality following major surgery remains a significant challenge for the National Health Service (NHS). The number of deaths identified each year by the National Confidential Enquiry into Peri-Operative Deaths (NCEPOD) changed little between 1989 and 2003 [1,2]. A recent analysis identified higher mortality rates in a UK hospital when compared with a similar institution in the USA [3]. Approximately 2.3 million surgical procedures are performed annually in the NHS, with an estimated mortality of 1.4% [4]. It is probable, however, that this low overall mortality rate conceals the existence of a subpopulation at much greater risk of postoperative complications and death. Successive NCEPOD reports show that the majority of deaths occur in older patients who undergo major surgery and who have severe coexisting disease [1,2]. The few available estimates suggest mortality rates of between 5.8% and 25% for this high-risk surgical population [5-11].

While the publication of surgeon-specific mortality data for cardiac surgery is a matter of public debate [12], other surgical specialties do not routinely collect such data. The NCEPOD provides information on those patients who die, but provides no indication of the size of the high-risk surgical population from which they are derived. Recent developments in perioperative care may significantly improve outcome for these patients [13,14]; however, in the absence of data describing the size of the high-risk population, it is difficult to convince clinicians and managers either of the need to introduce new therapeutic approaches or to provide additional resources for postoperative care. The aim of the present study was to ascertain what proportion of general surgical patients are at high risk of postoperative death.

## Materials and methods

Data were extracted from two large healthcare databases, one maintained by CHKS Ltd and the other maintained by the Intensive Care National Audit & Research Centre (ICNARC). CHKS provides comparative benchmarking services to NHS trusts. Data are created by a clerical coding method, similar to Hospital Episodes Statistics. Validation is performed locally by the Trust and centrally by CHKS Ltd to provide a quality-assured dataset that can be used to inform managerial and clinical decisions. The ICNARC case mix programme collects data on consecutive admissions to participating adult, general intensive care units (ICUs) in England, Wales and Northern Ireland. Data are collated locally by trained dedicated staff and are subject to local and central internal error checks [15].

Data were extracted on all adult surgical admissions to hospital (CHKS data) and to the ICU (ICNARC data) for 94 NHS hospitals in England, Wales and Northern Ireland between January 1999 and October 2004 inclusive. These hospitals were selected because they contributed to both databases throughout the study period. Admissions involving endoscopy, day-case surgery, cardiothoracic surgery, neurosurgery, organ transplantation, obstetrics or the surgical management of burns were excluded. For brevity, procedures that satisfied the inclusion criteria are described as general surgical procedures.

There are 6,920 surgical procedure codes in the Office of Population Censuses and Surveys (now part of Office for National Statistics and Surveys) classification. Surgical admissions to hospital were identified in the CHKS database by the presence of one of 4,910 codes that satisfied the inclusion criteria. Where more than one surgical procedure was performed during the same hospital admission, only the first procedure was included in the analysis. Several alternative Office of Population Censuses and Surveys codes may exist for any given procedure. In order to reduce bias arising from discrepancies in the coding process, procedures were categorised into one of 372 Healthcare Resource Groups (HRGs) based on clinical similarity and resource homogeneity. Many Office of Population Censuses and Surveys codes and HRG codes specify the presence of a complicating medical condition, the complexity of surgery or a particular age group. HRGs were then ranked according to mortality rates. High-risk surgical procedures were prospectively defined as those procedures included in an HRG with a mortality rate of 5% or more. The remaining procedures were classified as standard risk.

Surgical admissions to the ICU were identified in the ICNARC database by the source of admission (either operating theatre or operating theatre via ward), and were only included if the primary reason for admission was not an excluded surgical procedure. ICU admissions were prospectively divided into admissions directly to the ICU following surgery and admissions to the ICU following a period of postoperative care on a standard ward. Where patients were readmitted to the ICU, only the first admission was included in the analysis.

Data are presented as the median (interquartile range). Categorical data were tested with the chi-squared approximation, and continuous data were tested with the Mann–Whitney *U* test. Analysis was performed using GraphPad Prism version 4.0 (GraphPad Software, San Diego, CA, USA). Significance was set at *P *< 0.05.

## Results

### CHKS dataset

**Figure 1:**
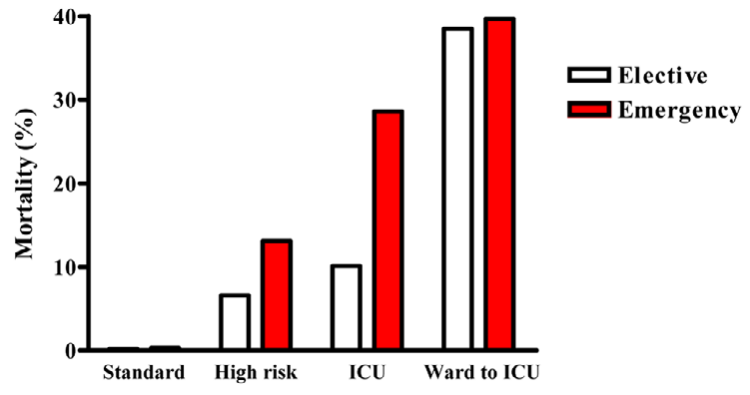
**Mortality rates for general surgical patients identified from the CHKS and ICNARC databases**. CHKS database: standard, all patients admitted to hospital for a general surgical procedure with an overall mortality rate of less than 5%; high risk, subpopulation of patients undergoing a procedure with an overall mortality rate of 5% or more. ICNARC database: ICU, general surgical patients admitted directly to the intensive care unit following surgery; ward to ICU, patients admitted to the intensive care unit following initial postoperative care on a standard ward.

**Figure 2:**
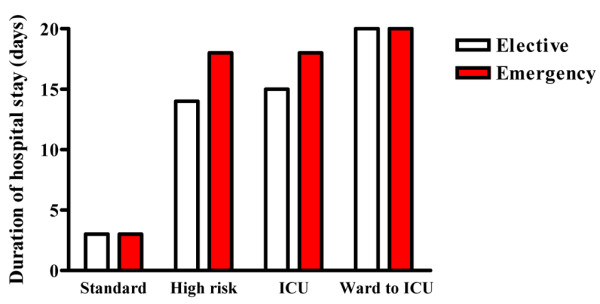
**Duration of hospital stay for general surgical patients identified from the CHKS and ICNARC databases**. CHKS database: standard, all patients admitted to hospital for a general surgical procedure with an overall mortality rate of less than 5%; high risk, subpopulation of patients undergoing a procedure with an overall mortality rate of 5% or more. ICNARC database: ICU, general surgical patients admitted directly to the intensive care unit following surgery; ward to ICU, patients admitted to the intensive care unit following initial postoperative care on a standard ward.

During the 70 months of the study, there were 4,117,727 hospital admissions involving a general surgical procedure, with 78,378 deaths (1.9%). The median age was 56 (39–71) years, and 1,784,909 patients were male (43%). There were 2,893,432 elective surgical admissions, with 12,704 deaths (0.44%), and 1,224,295 emergency admissions, with 65,674 deaths (5.4%) (Figure 1). The duration of the hospital stay was greater for emergency admissions than for elective admissions (5 (2–15) days versus 3 (1–6) days, *P *< 0.0001). The duration of hospital stay data for both datasets are presented in Figure 2.

Eighty-one out of 372 HRGs were associated with a mortality rate of 5% or greater. From these, 513,924 high-risk surgical procedures were identified, accounting for 83.8% of deaths but for only 12.5% of admissions (Table 1). Mortality rates were much greater in the high-risk population than in the standard population. Patients in the high-risk population were older, more likely to undergo emergency surgery and remained in hospital for prolonged periods. Complex or major surgery, advanced age, the presence of a complicating medical condition or a combination of these factors was specified by 51 of the 81 (63%) high-risk HRGs, compared with 95 of 291 (33%) standard-risk HRG codes. Mortality rates for a representative selection of HRG procedure codes are presented in Table 2.

### ICNARC dataset

Of 67,555 surgical admissions to the ICU, there were 59,424 general surgical admissions with 11,398 deaths (19%). Of these deaths, 4,653 (40.8%) occurred after initial discharge from the ICU; 3,529 patients were subsequently readmitted to the ICU, with 1,332 deaths (37.7%). The median age was 68.7 (56.3–76.8) years, and 35,156 patients were male (59.2%). There were 56,397 admissions directly to the ICU: 31,633 following elective surgery, with 3,199 deaths (10.1%), and 24,764 following emergency surgery, with 7,084 deaths (28.6%) (Figure 1). A further 3,027 patients were admitted to the ICU following initial postoperative care on a standard ward. Of these, 1,766 followed elective surgery, with 643 deaths (36.4%), and 1,261 followed emergency surgery, with 472 deaths (37.4%) (Figure 1).

For elective ICU admissions, the duration of the ICU stay was 1.1 (0.8–2.4) days and the duration of hospital stay was 15 (10–26) days. For emergency ICU admissions the duration of the ICU stay was 2.1 (0.9–5.6) days and the duration of the hospital stay was 18 (10–35) days (Figure 2). There were 3,283 early discharges from the ICU because of bed shortages (6.2%) but only 338 (0.7%) discharges from the ICU for palliative care. There were 7,807 discharges to high-dependency units that did not contribute data to the ICNARC database (14.8%).

## Discussion

This study confirms the existence of a large population of high-risk surgical patients with a hospital mortality rate of 12.3%. This population accounts for 83.8% of deaths but for only 12.5% of hospital admissions. Assuming the hospitals used in this analysis are representative of all the hospitals in the United Kingdom where general surgical procedures are performed, it is estimated that there are 1.3 million general surgical procedures per annum, with 25,000 deaths. Of these, 166,000 would be high-risk surgical procedures according to the definition used in this analysis. High mortality rates relate to advanced age, comorbidities and the complex nature of the surgery, which is often performed as an emergency.

Although these risk factors are well described [1,2], only a small proportion of this high-risk population was admitted to the ICU. Mortality rates among general surgical admissions to the ICU were higher still, and yet the duration of the ICU stay was short. It seems that patients were often discharged to the ward prematurely. Prolonged hospital stays occurred in both the overall high-risk population and in patients admitted to the ICU. This suggests that such patients have prolonged and complex medical needs. Among ICU patients more than 40% of deaths occur after initial discharge from the ICU, while less than 1% of ICU patients are discharged for palliative care. The observation that only 6.2% of patients were classified as having been discharged from the ICU prematurely suggests we are not able to identify those patients who require continued ICU care. The highest mortality rates were identified in the group of patients admitted to the ICU following initial care on a standard ward following surgery. The findings of this study are consistent with current mortality estimates for this population [1-11,16], and confirm the suggestion that the high-risk surgical population is much larger than previously thought.

Poor outcomes among the high-risk general surgical population are emphasised by comparison with cardiac surgery. Although cardiac surgical patients undergo major surgery and have a high incidence of coexisting disease, this population has an overall mortality rate of only 3.5% (excluding surgery for congenital heart disease) and a mortality rate of just 2.0% for patients undergoing coronary artery bypass grafting [17]. Several factors may account for this difference, but the availability of dedicated ICU facilities is likely to be of particular importance. While ICU admission following cardiac surgery is routine, provision of critical care facilities for major general surgery is limited. These findings emphasise the importance of recognising patients who are at high risk of postoperative complications and death, and ensuring they receive an appropriate level of postoperative care. This issue has also been highlighted by NCEPOD reports, which identify inadequate provision of ICU resources as a factor in postoperative death [1,2].

While the benefit of postoperative critical care admission may seem self-evident to some, others suggest this remains to be proven. Indeed, there is little evidence that the wider availability of critical care facilities improves the life expectancy of any large population. A recent study from North America, however, has explored the determinants of long-term survival following major surgery [18]. In a population of 105,000 surgical patients, the occurrence of complications within 30 days of major surgery was found to be a much more important determinant of long-term survival than either preoperative comorbidity or intraoperative adverse events. The authors of this report conclude that healthcare resources should therefore be focused on the prevention of complications. Recent developments in postoperative critical care suggest that a considerable reduction in postoperative complication rates may be possible [13,14].

There are limitations with the use of data extracted from large healthcare databases. In the absence of a more reliable system for estimating postoperative mortality rates, however, it is necessary to rely on data currently available. Difficulties with the use of Hospital Episodes Statistics and similar data in the identification of postoperative deaths are well recognised. In particular, the coding process is not designed to capture detailed mortality data, although this appears to result in an underestimate of mortality rates [1,2,17,19].

Data provided by CHKS are subject to similar limitations, but may be more accurate than those extracted from the Hospital Episodes Statistics database. NHS Trusts work together with CHKS to validate data, which are collated for the purpose of assessing Trust performance rather than to satisfy statutory requirements. Data extracted from the ICNARC database provide an accurate description of ICU admissions and resource use, although 14.8% of patients were discharged from the ICU to high-dependency units that did not contribute data to ICNARC. This observation suggests that ICNARC data may underestimate provision of critical care resources for surgical patients. This factor can, however, only account for a small proportion of the short fall in critical care resource provision for high-risk surgical patients. While there are fewer high-dependency unit beds than ICU beds in the United Kingdom [16], data from this study suggest that the number of high-risk procedures may be more than eight times the number of ICU admissions. It is possible that not all admissions identified in each database were drawn from the same population. Consequently, only a limited and cautious interpretation of the combined dataset has been performed.

## Conclusion

The present study confirms the existence of a large population of high-risk general surgical patients, which accounts for around 13% of surgical admissions but more than 80% of postoperative deaths. Only a small proportion of this population is admitted to the ICU, suggesting inadequate critical care resource provision. Better preoperative identification of these high-risk patients is required. Furthermore, an accurate system is needed to collect mortality data for all surgical specialties.

## Key messages

• 	The incidence of postoperative death in the United Kingdom has changed little in recent years. Most deaths occur in older patients, with coexisting medical disease, who undergo major surgery.

• 	Over 80% of postoperative deaths occur in a subpopulation of high-risk surgical patients.

• 	Fewer than 15% of these high-risk patients are admitted to intensive care following surgery.

## Abbreviations

HRG = Healthcare Resource Group; ICNARC = Intensive Care National Audit & Research Centre; ICU = intensive care unit; NCEPOD = National Confidential Enquiry into Peri-Operative Deaths; NHS = National Health Service.

## Competing interests

The authors declare that they have no competing interests.

## Authors' contributions

All authors were involved in data analysis and drafting the manuscript, and approved the final version. All authors had full access to data and take responsibility for the integrity of the data and the accuracy of the analysis.

## Figures and Tables

**Table 1:** Data for two populations of general surgical patients identified from the CHKS database

	Standard population	High-risk population	*P*
*n*	3,603,803	513,924	-
Age (years)	54 (38–69)	75 (63–83)	<0.0001
Emergency procedures	769,371 (21.3%)	454,924 (88.5%)	<0.0001
Duration of hospital stay (days)	3 (1–6)	16 (9–29)	<0.0001
Mortality	15,038 (0.42%)	63,340 (12.3%)	<0.0001

Data presented as median (interquartile range) or *n *(%). Standard population, all patients admitted to hospital undergoing a procedure with an overall mortality rate of less than 5%; high-risk population, patients undergoing a procedure with an overall mortality rate of 5% or greater.

**Table 2:** Mortality rates for selected Healthcare Resource Group procedure codes

Hospital Resource Group procedure code	*n*	Urgency	Deaths (*n*)	Mortality rate (%)
Q01: Emergency aortic surgery	6,598	Emergency	2,721	41.24
F33: Large intestine; major procedures with complicating condition(s)	5,765	Emergency	1,290	22.38
F41: General abdominal; very major or major procedures aged over 69 years or with complicating condition(s)	11,648	Emergency	1,843	15.82
H05: Complex hip or knee revisions	1,667	Elective	186	11.16
H33: Neck of femur fracture; aged over 69 years or with complicating condition(s)	170,804	Emergency	15,780	9.24
F11: Stomach or duodenum; complex procedures	3,714	Elective	312	8.40
Q02: Elective abdominal vascular surgery	17,791	Elective	1,321	7.43
F01: Oesophagus; complex procedures	5,594	Elective	375	6.70
F32: Large intestine; very major procedures	44,814	Elective	1,521	3.39
Q03: Lower limb arterial surgery	18,247	Elective	480	2.63
L02: Kidney major open procedure; aged over 49 years or with complicating condition	17,549	Elective	343	1.95
H02: Primary hip replacement	123,785	Elective	507	0.41
L27: Prostate transurethral resection; aged over 69 years or with complicating condition	6,196	Elective	24	0.39
B02: Phakoemulsification cataract extraction with lens implant	89,444	Elective	50	0.06
F82: Appendicectomy procedures; aged less than 70 years with no complicating condition	88,067	Emergency	15	0.02

Data extracted from CHKS database. Note that several Hospital Resource Group codes may exist for any given procedure; as a result, these data may not accurately describe mortality rates for a specific procedure.

## References

[B1] Campling EA, Devlin HB, Lunn JN (1990). Report of the National Confidential Enquiry into Peri-Operative Deaths.

[B2] Cullinane M, Gray AJ, Hargraves CM, Lansdown M, Martin IC, Schubert M (2003). The 2003 Report of the National Confidential Enquiry into Peri-Operative Deaths.

[B3] Bennett-Guerrero E, Hyam JA, Shaefi S, Prytherch DR, Sutton GL, Weaver PC, Mythen MG, Grocott MP, Parides MK (2003). Comparison of P-POSSUM risk-adjusted mortality rates after surgery between patients in the USA and the UK. Br J Surg.

[B4] (2000). Quality and Performance in the NHS: NHS Performance Indicators.

[B5] Tekkis PP, Poloniecki JD, Thompson MR, Stamatakis JD (2003). Operative mortality in colorectal cancer: prospective national study. BMJ.

[B6] McCulloch P, Ward J, Tekkis PP (2003). Mortality and morbidity in gastro-oesophageal cancer surgery: initial results of ASCOT multicentre prospective cohort study. BMJ.

[B7] Bradbury AW, Adam DJ, Makhdoomi KR, Stuart WP, Murie JA, Jenkins AM, Ruckley CV (1998). A 21-year experience of abdominal aortic aneurysm operations in Edinburgh. Br J Surg.

[B8] Catto JW, Alexander DJ (2002). Pancreatic debridement in a district general hospital – viable or vulnerable?. Ann R Coll Surg Engl.

[B9] Mella J, Biffin A, Radcliffe AG, Stamatakis JD, Steele RJ (1997). Population-based audit of colorectal cancer management in two UK health regions. Colorectal Cancer Working Group, Royal College of Surgeons of England Clinical Epidemiology and Audit Unit. Br J Surg.

[B10] Goldacre MJ, Roberts SE, Yeates D (2002). Mortality after admission to hospital with fractured neck of femur: database study. BMJ.

[B11] Bayly PJ, Matthews JN, Dobson PM, Price ML, Thomas DG (2001). In-hospital mortality from abdominal aortic surgery in Great Britain and Ireland: Vascular Anaesthesia Society audit. Br J Surg.

[B12] Treasure T (2005). Mortality in adult cardiac surgery. BMJ.

[B13] Squadrone V, Coha M, Cerutti E, Schellino MM, Biolino P, Occella P, Belloni G, Vilianis G, Fiore G, Cavallo F, Ranieri VM (2005). Continuous positive airway pressure for treatment of postoperative hypoxemia: a randomized controlled trial. JAMA.

[B14] Pearse RM, Dawson D, Fawcett J, Rhodes A, Grounds RM, Bennett ED (2005). Early goal-directed therapy after major surgery reduces complications and duration of hospital stay. A randomised, controlled trial. Crit Care.

[B15] Harrison D, Brady A, Rowan K (2004). Case mix, outcome and length of stay for admissions to adult, general critical care units in England, Wales and Northern Ireland: the Intensive Care National Audit & Research Centre Case Mix Programme Database. Crit Care.

[B16] Goldhill DR (2005). Preventing surgical deaths: critical care and intensive care outreach services in the postoperative period. Br J Anaesth.

[B17] Keogh BE, Kinsman R (2005). Fifth National Adult Cardiac Surgical Database Report.

[B18] Khuri SF, Henderson WG, DePalma RG, Mosca C, Healey NA, Kumbhani DJ (2005). Determinants of long-term survival after major surgery and the adverse effect of postoperative complications. Ann Surg.

[B19] Poloniecki JD, Roxburgh JC (2000). Performance data and the mortuary register. Ann R Coll Surg Engl.

